# Rapeseed (canola) oil aggravates metabolic syndrome-like conditions in male but not in female stroke-prone spontaneously hypertensive rats (SHRSP)

**DOI:** 10.1016/j.toxrep.2022.01.011

**Published:** 2022-02-07

**Authors:** Mai Nishikawa, Naoki Ohara, Yukiko Naito, Yoshiaki Saito, Chihiro Amma, Kenjiro Tatematsu, Jinhua Baoyindugurong, Daisuke Miyazawa, Yoko Hashimoto, Harumi Okuyama

**Affiliations:** a College of Pharmacy, Kinjo Gakuin University, 2-1723 Omori, Moriyama-ku, Nagoya, Aichi 463-8521, Japan; b School of Allied Health Sciences, Kitasato University, 1-15-1 Kitasato, Minami-ku, Sagamihara, Kanagawa 252-0373, Japan; c Hatano Research Institute, Food and Drug Safety Center, 729-5 Ochiai, Hadano, Kanagawa 257-8523, Japan; d Gifu Pharmaceutical University, 5-6-1 Mitabora, Gifu, Gifu 502-8585, Japan; e Inner Mongolia Agricultural University, College of Food Science and Engineering, Zhaowuda Rd. 306, Hohhot, Inner Mongolia 010018, PR China; f School of Dentistry, Aichi-Gakuin University, 1-100 Kusumoto-cho, Chikusa-ku, Nagoya, Aichi 464-8650, Japan

**Keywords:** AUC, area under the curve, BW, body weight, CAN, Canola oil, FC, food consumption, FCh, free cholesterol, FFA, free fatty acid, GAPDH, glyceraldehyde-3-phosphate dehydrogenase, Glu, glucose, MR, mineralocorticoid receptor, OGTT, oral glucose tolerance test, RAAS, renin-angiotensin-aldosterone system, SHR, spontaneously hypertensive rat, SHRSP, stroke-prone spontaneously hypertensive rat, SOY, soybean oil, TCh, total cholesterol, TG, triglyceride, WKY rat, Wistar Kyoto rat, Rapeseed (canola) oil, Life-shortening, SHRSP, Testosterone, Aldosterone, Sex difference

## Abstract

This study was conducted to investigate whether or not there are sex differences in canola oil (CAN)-induced adverse events in the rat and to understand the involvement and the role of testosterone in those events, including life-shortening. Stroke-prone spontaneously hypertensive rats (SHRSP) of both sexes were fed a diet containing 10 wt/wt% soybean oil (SOY, control) or CAN as the sole dietary fat. The survival of the males fed the CAN diet was significantly shorter than that of those fed the SOY diet. In contrast, the survival of the females was not affected by CAN. The males fed the CAN diet showed elevated blood pressure, thrombopenia and insulin-tolerance, which are major symptoms of metabolic syndrome, whereas such changes by the CAN diet were not found in the females. Plasma testosterone was significantly lower in animals of both sexes fed the CAN diet than in those fed the SOY diet, but interestingly, the lowered testosterone was accompanied by a marked increase in plasma aldosterone only in the males. These results demonstrate significant sex differences in CAN-toxicity and suggest that those sex differences may be attributable to the increased aldosterone level, which triggers aggravation of the genetic diseases specific to SHRSP, that is, metabolic syndrome-like conditions, but only in the males. The present results also suggest that testosterone may negatively regulate aldosterone production in the physiology of the males, and the inhibition of that negative regulation caused by the CAN diet is one of the possible causes of the adverse events.

## Highlights

• Canola oil shortens life of male SHRSP.
• Testis is the target of canola oil toxicity.
• Inhibition of negative regulation by testosterone of aldosterone production may be a trigger of canola oil toxicity.
• Facilitation of hypertension by aldosterone may lead to life-shortening.
• Increased plasma lipids by canola oil have no relevance to life-shortening.

## Graphical abstract

**Figure:**
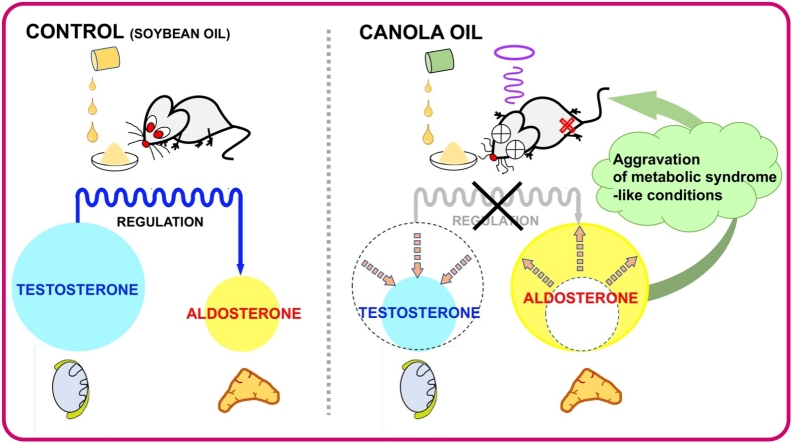

## 1 Introduction

Rapeseed oil (canola oil, CAN), corn oil and olive oil are consumed in quantity for cooking and food processing, but these oils have been reported to shorten unusually the survival of male stroke-prone spontaneously hypertensive rats (SHRSP) when one of these oils was fed as the sole or predominant dietary fat nutrient [[1], [2], [3], [4], [5]]. Although neither the causatives nor the underlying mechanisms for this life-shortening have yet been elucidated in detail, among the oils noted above, CAN is the one that has been investigated the most extensively for those effects in SHRSP [[6], [7], [8], [9], [10], [11]]. The major adverse events found in male SHRSP given a diet containing CAN are as follows: aggravated hypertension [7]; increased plasma lipids [8,9]; decreased antioxidant enzymes [10]; injury in the kidney [3]; and thrombopenia [8]. Previously, the advance of hypertension, accelerated onset of stroke and exacerbated vascular injuries in the kidney and heart were observed in male SHRSP after ingesting for 8 weeks a diet containing CAN, compared to animals fed a diet containing soybean oil (SOY), the control. However, no specific targets of CAN toxicity were identified on pathological examination [7]. On the other hand, significantly suppressed testosterone production was observed in male SHRSP given a CAN diet for 13 weeks, compared to animals given a SOY diet [12]. These findings imply that CAN ingestion promotes metabolic syndrome-like conditions by lowering the testosterone level in male SHRSP, since it is well known that testosterone deficiency is associated with metabolic syndrome in men and induces metabolic syndrome-like symptoms in rodents, as well [[13], [14], [15]]. In fact, the prevalence in men of metabolic syndrome is affected by androgen, and a decreased level of testosterone is connected with increased mortality in elderly men [16]. In particular, testosterone deficiency has been confirmed to cause insulin resistance, central obesity, dyslipidemia, hypertension and cardiovascular disease, which are the major symptoms of metabolic syndrome in men [17,18]. Among the experimental rat strains, SHRSP, which exhibits a predisposition to the development of complications similar to metabolic syndrome, has been studied as a helpful model animal for investigating metabolic syndrome in men [[19], [20], [21]]. Therefore, suppressed testosterone production could be one of the triggers for the early onset of metabolic syndrome-like symptoms and life-shortening observed in male SHRSP fed a CAN diet. The targets of CAN toxicity in SHRSP may include the organs producing sex hormones. It is unknown if CAN also decreases the testosterone level in men. Moreover, it is hard to imagine circumstances in which CAN would be the sole fat nutrient in a person’s diet. However, the adverse effects of CAN in SHRSP are worth investigating in more detail in relation to metabolic syndrome and differences in sex, considering those effects are induced by one of the most widely consumed oils in the dietary life of humans.

There are evident sex differences in the role of testosterone when the etiology of metabolic syndrome is examined in terms of the involvement of sex hormones. For instance, androgen deficiency is associated with a predisposition to metabolic syndrome in men, and testosterone replacement provides ameliorating effects [13]. In contrast, increased testosterone in postmenopausal women is associated with a prevalence of the disease [22]. So, to understand CAN toxicity from the perspective of it being a promoting factor for metabolic syndrome, the investigation of sex differences in CAN effects is critical. However, despite possible sex differences, almost all knowledge regarding the effects of CAN has been gained through research on male animals, and relatively little is known of the effects of a CAN diet on female SHRSP.

In the present study, the influences of a CAN diet, not only on life expectancy but also on several parameters which change in metabolic syndrome (*i.e.*, blood pressure, plasma lipids, platelet count, glucose (Glu) uptake and tissue injury in organs), were investigated and compared using SHRSP of both sexes. Additionally, it was found for the first time that the decreased plasma testosterone caused by a CAN diet was accompanied by a significant increase in plasma aldosterone in males. The possible involvement of aldosterone in CAN-induced adverse effects is also discussed.

## 2 Materials and methods

### 2.1 Diets

A fat-free AIN-93 G powder diet supplemented with 10 wt/wt% (24.8 energy percentage) soybean oil (SOY) and the same diet containing, instead, 10 wt/wt% CAN were purchased from CLEA Japan (Tokyo). The fatty acid compositions of the diets are shown in Table 1. SOY and CAN were gifts from the Japan Oilseed Processors Association (Tokyo).

**Table 1:** Fatty acid compositions (%) of diets.

Fatty acids		SOY	CAN
14 : 0	Myristic acid	0.14	0.22
16 : 0	Palmitic acid	9.01	4.37
16 : 1	Palmitoleic acid	0.07	0.21
18 : 0	Stearic acid	4.38	1.93
18 : 1	Oleic acid	23.64	63.67
18 : 2 n-6	Linoleic acid	54.50	19.56
18 : 3 n-3	Linolenic acid	7.23	8.00
20 : 0	Arachidic acid	0.39	0.59
20 : 1	Eicosenoic acid	0.20	1.15
22 : 0	Behenic acid	0.45	0.32
	n-6 / n-3	7.54	2.45
	Total fatty acids (g/100 g diet)	9.88	9.22

SOY, 10 w/w% soybean oil diet (control); CAN, 10 w/w% canola oil diet.

### 2.2 Survival experiment

#### 2.2.1 Animal husbandry

Forty-eight stroke-prone spontaneously hypertensive rats, SHRSP (Izm), 24 of each sex, at 4 weeks of age were purchased from Japan SLC (Shizuoka). Three animals were housed per plastic cage with a bedding of wood chips and acclimatized for 7 days in a room conditioned at a temperature of 23 ± 1 °C and a relative humidity of 50 ± 5 %, and with lighting from 8:00 to 20:00. After acclimation, the animals of each sex were divided into 2 groups of 12 animals each, and they were housed individually. The observation periods in the survival experiment were 471 days and 516 days for male and female animals, respectively. The animals in each group were given, *ad libitum*, the SOY diet or the CAN diet and tap water. The diets were discarded and replenished with fresh ones twice a week. The SOY diet was used as the control, since general rat chows contain SOY as the major fat ingredient, and the lifespan of SHRSP fed a SOY diet is in the mid-range of that of SHRSP fed one of several different diets, each of which contained a single oil or fat as the sole dietary fat [1]. In almost all of the previous studies, 1 %NaCl was added to drinking water to facilitate the elevation of blood pressure. In the present study, however, tap water was given and the animals without salt loading were kept under physiologically more normal conditions.

All the animals were used following the requirements set by the Committee for Ethical Usage of Experimental Animals in Kinjo Gakuin University.

#### 2.2.2 Gross observation, measurement of body weight and food consumption

The animals were weighed on the day before starting the administration and once a week thereafter. Food consumption (FC) was also measured once a week. All the animals were observed at least once daily for general condition. The following symptoms were regarded as signs of stroke: exophthalmos, hyperirritability, hyperkinesia, hyperresponsiveness, motion disturbance, tremor, convulsion, limb paralysis or sudden death [23,24]. When animals with severe behavioral disorders, total debilitation or eating disorders owing to stroke-related conditions were found, they were euthanized.

#### 2.2.3 Measurement of platelet count

A blood sample was obtained by a puncture of the caudal vein from every animal of both sexes during the 8th week, and from the female animals during the 16th week, as well. The platelet count in 10 μL of blood was determined by a hemocyte counter (MEK-5208, Nihon Kohden, Tokyo, Japan). Blood sampling at the 16th week was not performed in the male animals, since the mean body weight (BW) in the CAN group had decreased, and one animal had already died of stroke.

#### 2.2.4 Pathological examination

Postmortem examination was carried out on every animal as soon as it was found dead. The brain, heart, lung, spleen, liver, kidney, adrenal gland, testis, epididymis, ovary and uterus were removed, weighed and macroscopically examined. The organs were, then, immersed in 0.1 M phosphate buffer containing 10 % formalin. The organs with such severe autolysis that they were not suitable for pathological examination were omitted. The fixed organs or tissues were embedded in paraffin and sectioned, stained with hematoxylin and eosin, and examined under a microscope.

### 2.3 Eight-week feeding study

#### 2.3.1 Animal husbandry

Forty SHRSP (Izm), 20 of each sex and 4 weeks of age (Japan SLC, Shizuoka) were acclimatized for 7 days. Ten rats of each sex were assigned to 2 groups and given, *ad libitum*, the SOY diet or the CAN diet, and tap water for 8 weeks. The animals were maintained in an environment similar to that in the survival experiment, although 2 or 3 animals were housed per plastic cage, with a bedding of wood chips, in this experiment.

#### 2.3.2 Measurement of blood pressure and heart rate

During the 8th week of feeding, arterial blood pressure was measured with a tail-cuff, and changes in blood pressure and pulse rate were measured in every animal by a plethysmograph (MK-2000ST, Muromachi, Tokyo, Japan).

#### 2.3.3 Oral glucose tolerance test

During the 8th week of feeding, the animals were fasted for 18 h preceding an oral Glu tolerance test (OGTT). A sample of blood was taken from the caudal vein for the determination of the pre-load (control) Glu concentration, and then, the animals were given 3 g/kg of Glu by gavage. Thereafter, blood was taken every 30 min up to 150 min after Glu loading. One drop of each blood sample was used for determination of plasma Glu concentration using a coulometric detector for oxidization of Glu to gluconolactone, using a FreeStyle Freedom Lite™ (Nipro, Osaka). The rest of the blood was left for 30 min at room temperature, and then, it was centrifuged at 1200 × *g* for 30 min. The sera obtained were used for determination of insulin concentration using an ELISA kit (Ultra Sensitive Rat Insulin kit™, Morinaga Institute of Biological Science, Yokohama, Japan), following the instructions of the kit.

#### 2.3.4 Autopsy, blood biochemistry and determination of steroids

At the end of the feeding period, the animals were fasted for 18 h preceding autopsy. The animals were then anesthetized by sevoflurane (Fujifilm Wako, Osaka, Japan) inhalation, and a mid-line abdominal incision was made. Blood was drawn from the abdominal vein using heparin sodium (Nacalai Tesque, Kyoto, Japan) as an anticoagulant and centrifuged at 150 x g for 15 min at 4 °C. The plasma samples obtained were kept at -80 °C until use. After the blood sampling, the organs were removed, weighed and macroscopically examined. The left kidneys from the 6 animals in each group were frozen in liquid nitrogen and then stored at -80 °C for RNA extraction. The other organs were fixed in 0.1 M phosphate buffer containing 10 % formalin, embedded in paraffin, sectioned, stained and examined under a microscope.

Plasma concentrations of Glu, free fatty acid (FFA), triglyceride (TG), total cholesterol (TCh) and free cholesterol (FCh) were measured using commercially available kits: Glucose C-II test™, NEFA C-test™, Triglyceride E-test™, Cholesterol E-test™ and Free cholesterol E-test™ (Fujifilm Wako Pure Chemical, Osaka), respectively.

Plasma concentrations of steroids were determined using samples from the 6 animals in each group whose kidneys were used for the determination of mRNA expression for renin. The concentrations of steroids were determined using a method described previously [25]. Briefly, 2H- or 13C-labelled steroids were added to the plasma as internal standards, and steroids were extracted in diethyl ether. The standards used were as follows: testosterone-13C3 and aldosterone-d7 (IsoSciences, King of Prussia, PA, USA). The extracts were then purified using an InertSep pharma column (GL sciences, Tokyo, Japan) and then derivatized with picolinyl ester. The resulting derivatives were placed in an InertSep SI column (GL sciences), and the eluted fractions were injected into liquid chromatography-mass spectrometry/mass spectrometry (LC/MS/MS) equipment, comprising a Nexera UHPLC system (Shimadzu Co. Ltd., Kyoto, Japan) and an API4000 mass spectrometer (SCIEX, Framingham, MA, USA).

#### 2.3.5 Quantitative analysis of mRNA expression

Total RNA was extracted from the renal cortex tissue of the frozen kidney using Trizol™reagent (Thermo Fisher Scientific, Tokyo, Japan) and chloroform-isopropanol with phase-separation by centrifugation. The concentration of total RNA obtained was determined by the absorbance at 260 nm using an Ultra-micro spectrophotometer NanoDrop™ 1000 (Thermo Fisher Scientific). Two μg of total RNA was reverse-transcribed to cDNA using a High Capacity RNA-to cDNA™ kit (Thermo Fisher Scientific). The cDNA obtained was diluted (1:2) and used with EagleTaq universal MMX™ (Roche Diagnostics, Tokyo, Japan) and a probe/primer kit, TaqMan Assay™, specific for rat renin (Rn00561847_m1, Thermo Fisher Scientific) or for rat-glyceraldehyde-3-phosphate dehydrogenase (Rn01775763_g1, Thermo Fisher Scientific). Real-time PCR was performed using a 7300 Real-Time PCR System (Applied Biosystems-Thermo Fisher Scientific, Tokyo, Japan) programmed as follows: a 10-min holding period at 95 °C, followed by 40-cycle amplification for 15 s at 95 °C, and 1-min at 60 °C. Expression values were calculated according to the ΔΔCT method. The expression of mRNA for renin relative to that for glyceraldehyde-3-phosphate dehydrogenase (GAPDH) was compared between sexes and the 2 diets.

#### 2.3.6 Determination of plasma renin and angiotensin II

Plasma renin and angiotensin II concentrations were determined in triplicate using, respectively, a Rat Renin-1 ELISA kit (Raybiotech-Cosmo Bio, Tokyo, Japan), with a detection range of 27.4−20,000 pg/mL, and an Angiotensin II ELISA kit (Enzo Life Sciences-Cosmo Bio, Tokyo, Japan), with a detection range of 3.9–10,000 pg/mL. The ELISA procedures were performed following the instructions provided in the kits, and the results were obtained by subjecting 96-well plates to colorimetric detection at 450 nm.

### 2.4 Statistical methods

The differences in the incidence of pathological findings between sexes or between the dietary groups were evaluated by Fisher’s exact probability test (in Table 5). All other results are presented as means ± SEMs. The differences of the variables between sexes and between the diets given were compared by two way ANOVA (factors of sex and diet). Tukey’s multiple comparison was carried out when the interaction between the factors was significant or when considered advisable. In addition, the differences between the mean values of 2 dietary groups in each sex were also compared by unpaired *t*-test. In all cases, significance was set at p < 0.05. Prism v.9.10 (GraphPad Software, San Diego) was used for the statistical analyses.

## 3 Results

### 3.1 Survival experiment

#### 3.1.1 BW gain and FC

**Fig. 1:**
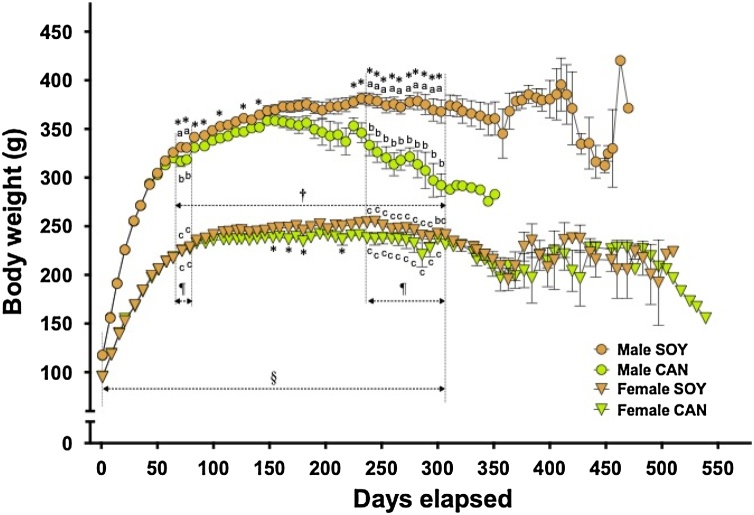
Body weight gain in SHRSP during the survival experiment. Twelve animals of each sex were assigned to the group fed 10 % soybean oil diet (SOY, control) or 10 % canola oil diet (CAN). Symbols with bars represent means with SEMs. ^§^ and ^†^, Significantly different between sexes and between the 2 different diets, respectively (p<0.05, two way ANOVA). ^¶^, Interaction exists between the factors, sexes and diets (p<0.05, two way ANOVA). Identical alphabetical letters indicate the absence of significant differences between the groups (p>0.05, Tukey’s test). *, Significantly different from the SOY group in each sex (p<0.05, unpaired *t*-test).

Changes in BW are shown in Fig. 1. Two way ANOVA analysis was performed at each time point. BW was consistently greater in the males than in the females. From the 70th day to the 308th day during the feeding period, BW of the animals given the CAN diet was significantly lower than that of those given the SOY diet regardless of sex (two way ANOVA). In the males, BW in the CAN group was significantly lower than in the SOY group on the 70th, 77th and 238th-308th days (Tukey’s test and unpaired *t*-test). In addition, significant differences between the dietary groups were found sporadically in both sexes on several days (unpaired *t*-test). The difference between the dietary groups gradually became larger in both sexes and was particularly evident in the males. Statistical analyses were not carried out on the 315th day and thereafter because only a small number of male animals remained alive in the CAN group. On the 168th day and thereafter in the males, and on the 350th day and thereafter in the females, BW varied as the number of animals with stroke increased, regardless of the diet given.

**Fig. 2:**
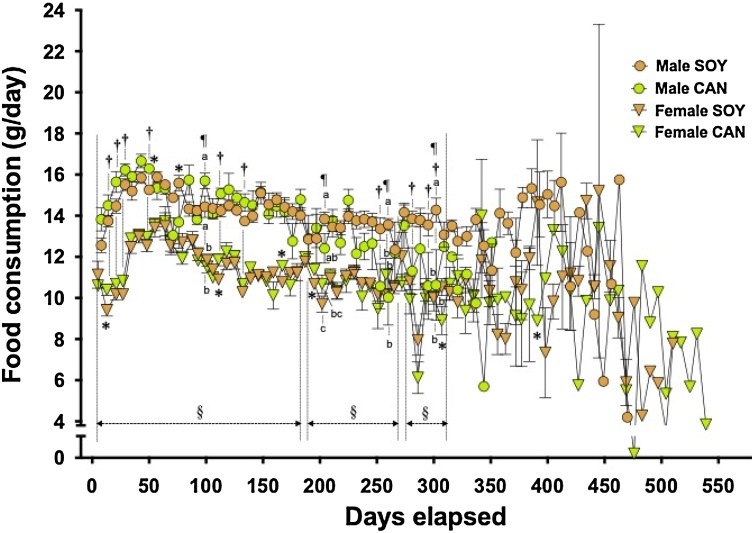
Food consumption in SHRSP during the survival experiment. Twelve animals of each sex were assigned to the group fed 10 % soybean oil diet (SOY, control) or 10 % canola oil diet (CAN). Symbols with bars represent means with SEMs. ^§^ and ^†^, Significantly different between sexes and between the 2 different diets, respectively (p<0.05, two way ANOVA). ^¶^, Interaction exists between the factors, sexes and diets (p<0.05, two way ANOVA). Identical alphabetical letters indicate the absence of significant differences between the groups (p>0.05, Tukey’s test). *, Significantly different from the SOY group in each sex (p<0.05, unpaired *t*-test).

Changes in FC are shown in Fig. 2. FC was also consistently greater in the males than in the females. Up to the 133rd day, FC in the animals given the CAN diet tended to be greater than in those given the SOY diet, and the difference was significant on the 14th, 21st, 28th, 49th, 112th and 133rd days (two way ANOVA). On the 140th day and thereafter, FC varied as the number of animals with stroke increased. A difference between the animals given the two diets could not be found, except on the 252nd, 280th, 294th and 301st days, when FC in the animals given the CAN diet was significantly less than in those given the SOY diet (two way ANOVA). On the 203rd, 259th and 301st days, FC in the males given the CAN diet was significantly less than in those given the SOY diet (Tukey’s test). In addition, significant differences between the dietary groups were found sporadically in both sexes (unpaired *t*-test).

#### 3.1.2 Survival time

**Fig. 3:**
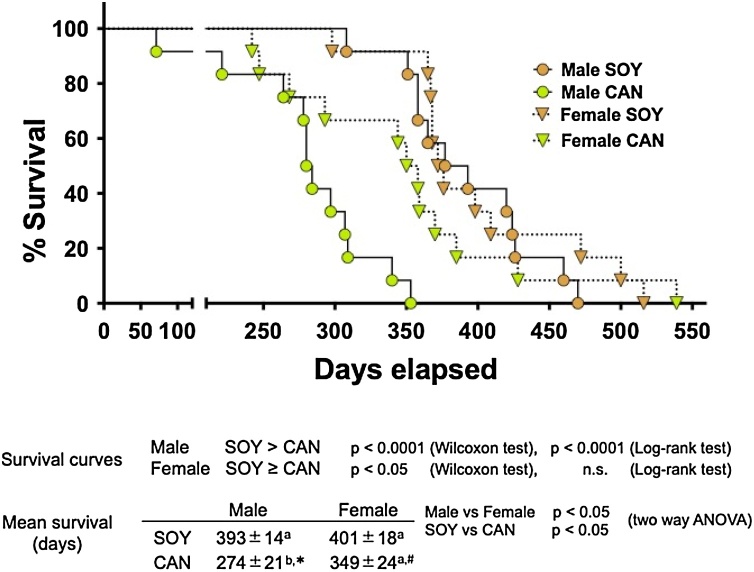
Survival curves of SHRSP fed 10 % soybean oil diet or 10 % canola oil diet. Twelve animals of each sex were assigned to the group given 10 % soybean oil diet (SOY, control) or 10 % canola oil diet (CAN). The survival curves in the males in the SOY group and in the CAN group were significantly different (Wilcoxon and log-rank tests). The curves in the females were evaluated significantly different between the 2 dietary groups by Wilcoxon test but not by log-lank test. The mean survival time was significantly different between sexes, female>male, and between the animals given the 2 different diets, SOY>CAN (two way ANOVA). Identical alphabetical letters indicate the absence of significant differences between the groups (p>0.05, Tukey’s test). *, Significantly different from the SOY group in the males (p<0.05, unpaired *t*-test). ^#^, Significantly different from the male animals given CAN diet (p<0.05, unpaired *t*-test).

As shown in Fig. 3, the survival curves for the males in the SOY and CAN groups were significantly different (Wilcoxon and log-rank tests). In contrast, the curves for the females did not differ as much as those for the males, except in the early phase, until the 4th death in the CAN group, at which point, in the CAN group, the female curve became similar to the male curve. The curves for the females were evaluated as significantly different between the 2 dietary groups by Wilcoxon test but not by log-rank test. The mean survival time was significantly different between the sexes, female > male, and the mean survival times of the animals given the SOY diet and those of the animals given the CAN diet were significantly different, SOY > CAN (two way ANOVA). In the males, the mean survival time in the CAN group was significantly shorter than in the SOY group. In contrast, the survival time of the females was not different between the 2 dietary groups (Tukey’s test and unpaired *t*-test). In the males, the mean onset time for stroke-related symptoms in the CAN group, 128 ± 15 days, was significantly shorter than in the SOY group, 214 ± 21 days (unpaired *t*-test). However, in the females the onset time in the SOY group, 296 ± 13 days was not significantly different from that in the CAN group, 249 ± 24 days (unpaired *t*-test). There was no significant sex difference in the mean survival time in the SOY groups, 393 ± 14 days in the males and 401 ± 18 days in the females (unpaired *t*-test). However, comparing the CAN groups, the mean survival time of the males, 274 ± 21 days, was significantly shorter than that of the females, 349 ± 24 days (unpaired *t*-test).

#### 3.1.3 Platelet count　　

**Fig. 4:**
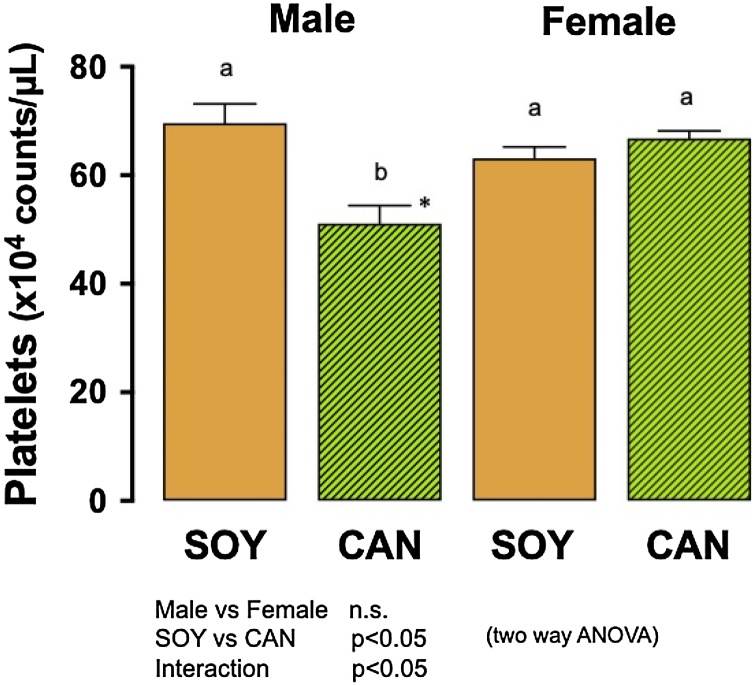
Platelet count in SHRSP fed 10 % soybean oil diet (SOY, control) or 10 % canola oil diet (CAN). At the 8th week of feeding during the survival experiment, platelet count was determined. Columns with bars represent means with SEMs of 12 animals. Platelet count was similar in both sexes but was significantly different between the animals given the 2 different diets (p<0.05, two way ANOVA). Platelet count in the males in the CAN group was significantly lower than any other group. Identical alphabetical letters indicate the absence of significant differences between the groups (p>0.05, Tukey’s test). *, Significantly different from the SOY group in the males (p<0.05, unpaired *t*-test).

Fig. 4 shows platelet counts at the 8th week during the survival experiment. Platelet counts were similar in the animals of both sexes but were significantly different between the animals given the SOY diet and those given the CAN diet (two way ANOVA). In the males, the platelet count in the CAN group was significantly lower than in the SOY group (Tukey’s test and unpaired *t*-test). In contrast, platelet counts in the females were similar in the 2 dietary groups. The platelet count at the 16th week was measured only in the females and was similar in the SOY group, 51.2 ± 1.8 × 10^4^ /μL, and in the CAN group, 55.3 ± 2.0 × 10^4^ /μL (unpaired *t*-test).

#### 3.1.4 Postmortem examinations

##### 3.1.4.1 Organ weight and macroscopic findings

Significant sex differences were found in absolute organ weights of the heart, lung, liver and kidney. The absolute weights of these organs were heavier in the males than in the females. Significant sex differences were also found in relative organ weights of the brain, spleen, kidney and adrenal gland. The relative weights of these organs were heavier in the females than in the males. Neither the absolute nor the relative weight of any organ showed significant differences attributable to the 2 different diets given (two way ANOVA, data not shown). There were no differences in the absolute or relative weights of any organs between the 2 dietary groups in either sex, except the absolute weight of the ovary (unpaired *t*-test, data not shown). The ovary weight in the CAN group, 77.6 ± 4.7 mg, was significantly greater than in the SOY group, 59.8 ± 5.6 mg. However, the relative weights were comparable, 0.41 ± 0.04 mg/g and 0.46 ± 0.04 mg/g, in the SOY group and the CAN group, respectively (unpaired *t*-test). Most of the animals showed hemorrhaging in the brain, hypertrophy in the heart, congestion in the lung and the liver, and fibrosis in the kidney. There were no differences in the frequencies of abnormal findings in any organ between the 2 dietary groups of either sex (Fisher’s exact probability test, data not shown).

##### 3.1.4.2 Microscopic findings

Cerebral hemorrhaging found on macroscopic examination was microscopically confirmed. Ventricular dilation, thrombosis and edema were observed in the brain. Myocardial hypertrophy and fibrosis were found in all the animals. Coronary arteriosclerosis and chronic nephrosis with thickened arterial walls were also found in almost all the animals. Hardly any differences were found in the frequencies of abnormalities in any organ between the groups or between sexes (Fisher’s exact probability test, data not shown).

### 3.2 Eight-week feeding study

#### 3.2.1 Blood pressure and heart rate

Table 2 shows changes in blood pressure and heart rate. Both, sex differences and dietary-differences, were found in blood pressure (two way ANOVA). Blood pressure in the males was higher than in the females. The significant dietary-difference by two way ANOVA reflected a marked increase by the CAN diet of blood pressure in the males. Systolic blood pressure, diastolic blood pressure and heart rate in the males were significantly increased in the CAN group, compared to those in the SOY group (Tukey’s test and unpaired *t*-test). In contrast, neither blood pressure nor heart rate in the females was different between the 2 dietary groups.

**Table 2:** Blood pressure and heart rate in SHRSP of both sexes given SOY diet or CAN diet for 8 weeks.

Parameters	Male						Female					
	SOY			CAN			SOY			CAN		
Systolic BP^§,†,¶^	230	±	5^b ^	267	±	7^a,*^	209	±	4^c^	207	±	4^c^
(mmHg)												
Diastolic BP^§,†,¶^	168	±	7^b^	197	±	6^a,*^	152	±	4^b^	148	±	4^b^
(mmHg)												
Heart rate^¶^	470	±	11^b^	501	±	7^a,*^	488	±	5^ab^	473	±	7^b^
(beats/min)												

SOY, 10 w/w% soybean oil diet (control); CAN, 10 w/w% canola oil diet. BP, blood pressure.

Values are means ± SEMs of 10 animals.

^§^ and ^†^, Significantly different between sexes and between diets, respectively (p<0.05, two way ANOVA).

^¶^, Interaction exists between the factors, sexes and diets (p<0.05, two way ANOVA).

Identical alphabetical letters (above SEMs) indicate the absence of significant differences between the groups (p>0.05, Tukey’s test).

*, Significantly different from the SOY group in each sex (p<0.05, unpaired *t*-test).

#### 3.2.2 OGTT

**Fig. 5:**
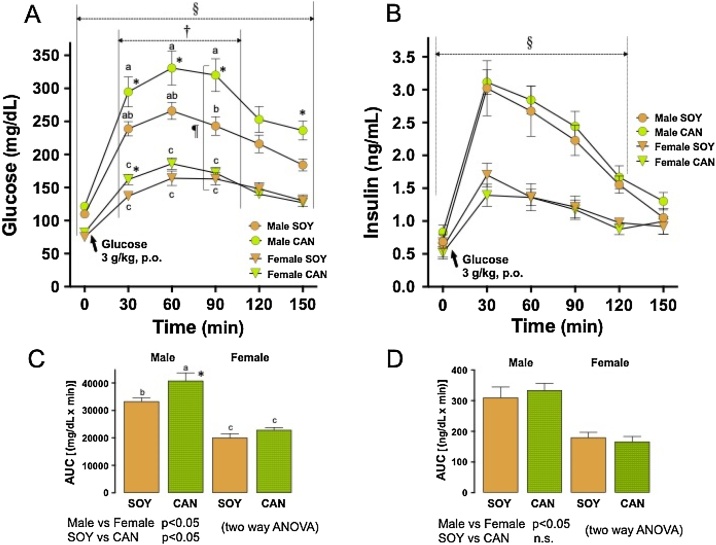
Oral glucose tolerance test (OGTT) in SHRSP fed 10 % soybean oil diet (SOY, control) or 10 % canola oil diet (CAN) for 8 weeks. Symbols or columns with bars represent means with SEMs of 10 animals. A. Time course of plasma Glu, B. Time course of serum insulin; ^§^ and ^†^, Significantly different between sexes and between the 2 different diets, respectively (p<0.05, two way ANOVA). ^¶^, Interaction exists between the factors, sexes and diets (p<0.05, two way ANOVA). Identical alphabetical letters indicate the absence of significant differences between the groups (p>0.05, Tukey’s test). *, Significantly different from the SOY group in each sex (p<0.05, unpaired *t*-test). C. AUCs for the curves of Glu levels; The AUCs were significantly different between sexes and between the 2 diets given (two way ANOVA). Identical alphabetical letters indicate the absence of significant differences between the groups (p>0.05, Tukey’s test). *, Significantly different from the SOY group in the males (unpaired *t*-test). D. AUCs for the curves of insulin levels. The AUCs were significantly different between sexes but not different between the 2 different diets (two way ANOVA).

The time courses for plasma Glu and serum insulin concentrations are shown with column bar graphs of the respective areas under the curves (AUCs) in Fig. 5. Significant sex differences were found in Glu concentration during 150 min after the Glu loading. Glu concentrations in the males were consistently greater than in the females. Glu concentrations in the animals given the CAN diet were significantly different from those in the animals given the SOY diet at 30, 60 and 90 min after Glu loading. That is, the CAN diet increased plasma glucose concentrations (two way ANOVA, Fig. 5A). Especially in the males, Glu concentrations in the CAN group were evidently increased and were significantly higher than in the SOY group at 30, 60, 90 and 150 min after Glu loading (Tukey’s test and unpaired *t*-test). In the females, Glu concentrations in the CAN group were significantly higher than in the SOY group at 30 min after Glu loading, but the differences were very slight (unpaired *t*-test). The AUCs for the time courses of Glu level were significantly different between the sexes and between the animals given the 2 different diets (two way ANOVA, Fig. 5C). In the males, the AUC in the CAN group was greater than that in the SOY group. In contrast, the AUCs were similar in the females regardless of the diets given (Tukey’s test and unpaired *t*-test, Fig. 5C).

Significant sex differences were also found in serum insulin concentration during120 min after Glu loading. Insulin concentration in the males was consistently higher than in the females. Insulin concentrations in the animals given the 2 different diets were similar regardless of sex (two way ANOVA, Fig. 5B). The AUCs for the time courses of insulin level also showed sex differences, while the AUCs were similar between the animals given the 2 different diets (two way ANOVA, Fig. 5D). In both sexes, no significant differences were found between the dietary groups.

#### 3.2.3 Plasma Glu and lipids

Plasma Glu and lipid concentrations on autopsy are shown in Table 3. All the parameters, except FFA, showed significant sex differences (two way ANOVA). The Glu concentration in the males was higher than in the females. The TCh, FCh and TG concentrations in the females were higher than in the males. The concentrations of all 3 lipids were significantly different between the animals given the 2 different diets (two way ANOVA). The TCh, FCh and TG concentrations in the CAN group were significantly higher than in the SOY group in both sexes (Tukey’s test and unpaired *t*-test).

**Table 3:** Plasma glucose and lipid concentrations in SHRSP of both sexes given SOY diet or CAN diet for 8 weeks.

Parameters	Male						Female					
	SOY			CAN			SOY			CAN		
Glucose^§^	144	±	4.7^ab^	155	±	2.7^a^	135	±	4.0^b^	137	±	1.9^b^
(mg/dL)												
Total cholesterol^§,†^	56.0	±	7.1^b^	76.2	±	5.3^ab,*^	64.9	±	4.1^b^	92.0	±	4.0^a,*^
(mg/dL)												
Free cholesterol^§,†^	8.28	±	0.7^d^	15.1	±	0.9^c,*^	18.1	±	0.7^b^	22.9	±	0.5^a,*^
(mg/dL)												
Triglyceride^§,†^	67.7	±	3.3^c^	107	±	8.6^b,*^	123	±	8.8^ab^	148	±	5.9^a,*^
(mg/dL)												
Free fatty acids	1.05	±	0.1	0.97	±	0.1	0.89	±	0.1	0.90	±	0.1
(mEq/L)												

SOY, 10 w/w% soybean oil diet (control); CAN, 10 w/w% canola oil diet.

Values are means ± SEMs of 10 animals.

§ and †, Significantly different between sexes and between diets, respectively (p<0.05, two way ANOVA).

Identical alphabetical letters (above SEMs) indicate the absence of significant differences between the groups (p>0.05, Tukey’s test).

*, Significantly different from the SOY group in each sex (p<0.05, unpaired *t*-test).

#### 3.2.4 Plasma steroids

**Fig. 6:**
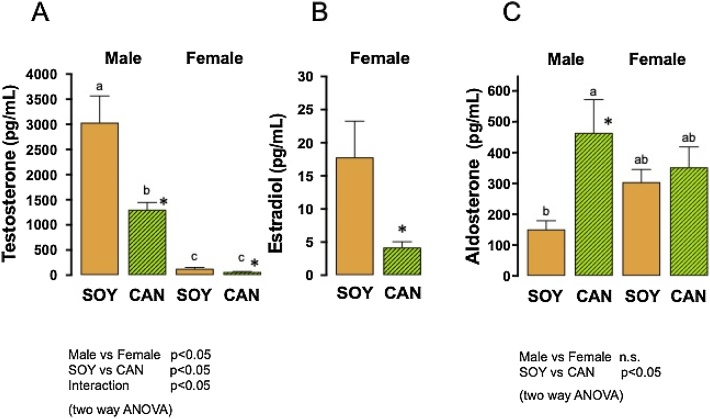
Plasma testosterone, estradiol and aldosterone levels in SHRSP fed 10 % soybean oil diet (SOY, control) or 10 % canola oil diet (CAN) for 8 weeks. Columns with bars represent the means with SEMs of 6 animals. A. Plasma testosterone concentration was significantly different between sexes and between the 2 different diets (p<0.05, two way ANOVA). Identical alphabetical letters indicate the absence of significant differences between the groups (p>0.05, Tukey’s test). *, Significantly different from the SOY group in each sex (p<0.05, unpaired *t*-test). Testosterone level in the males in the CAN group was significantly lower than in the SOY group (unpaired *t*-test). In the females, testosterone level in the CAN group was also significantly lower than in the SOY group (unpaired *t*-test). B. In the females, the CAN diet-induced decrease in testosterone was associated with a significantly lower estradiol level in the CAN group than in the SOY group (unpaired *t*-test). C. Aldosterone level was not different between sexes but significantly different between the 2 different diets (two way ANOVA). In the males, the aldosterone level in the CAN group was significantly higher than in the SOY group (Tukey’s test and unpaired *t*-test). Aldosterone concentrations in the females did not show difference between the 2 different diets.

As shown in Fig. 6A, plasma testosterone concentration was significantly different between sexes and between the animals given the 2 different diets. Testosterone concentration was significantly higher in the males than in the females and was decreased by the CAN diet regardless of sex (two way ANOVA). In the males, testosterone concentration in the CAN group, 1302 ± 143 pg/mL, was significantly lower than in the SOY group, 3034 ± 530 pg/mL (Tukey’s test and unpaired *t*-test). In the females, testosterone concentration in the CAN group, 62.7 ± 8.7 pg/mL, was also lower than in the SOY group, 128.4 ± 24.7 pg/mL (unpaired *t*-test). The CAN diet-induced decrease in testosterone in the females might be associated with the significantly lower estradiol concentration in the CAN group, 4.2 ± 0.9 pg/mL, than in the SOY group, 17.8 ± 5.6 pg/mL (unpaired *t*-test, Fig. 6B). The aldosterone concentration did not differ between sexes but differed significantly between the animals given the 2 different diets. The animals given the CAN diet showed a significantly increased aldosterone concentration, compared to the animals given the SOY diet (two way ANOVA). In the males, the aldosterone concentration in the CAN group, 464.1 ± 107.8 pg/mL, was significantly (3 times) greater than in the SOY group, 151.2 ± 27.9 pg/mL. In contrast, the aldosterone concentration in the females was increased slightly by CAN (*i.e.*, 352.0 ± 66.3 pg/mL) but was not significantly different from that in those given the SOY diet (*i.e.*, 304.3 ± 41.0 pg/mL) (Tukey’s test and unpaired *t*-test, Fig. 6C).

#### 3.2.5 Expression of mRNA for renin

**Fig. 7:**
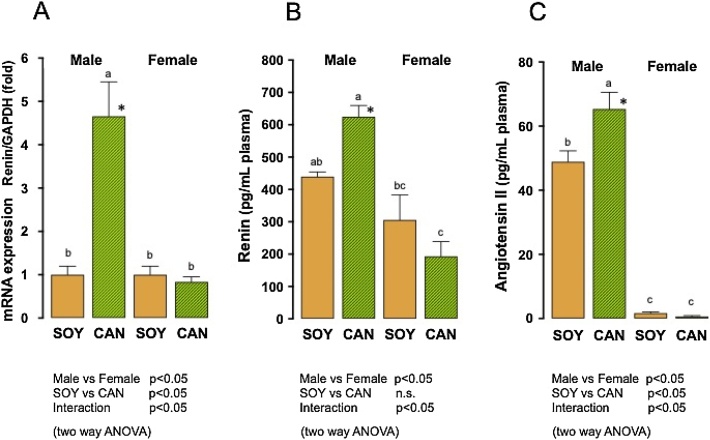
Expression of mRNA for renin in the kidney and plasma concentrations of renin and angiotensin II in SHRSP fed 10 % soybean oil diet (SOY, control) or 10 % canola oil diet (CAN) for 8 weeks. Columns with bars represent the means with SEMs of 6 animals. Identical alphabetical letters indicate the absence of significant differences between the groups (p>0.05, Tukey’s test). *, Significantly different from the SOY group in each sex (p<0.05, unpaired *t*-test). A. Expression of mRNA for renin/that for GAPDH was significantly different between sexes and between the 2 different diets (p<0.05, two way ANOVA). The gene expression for renin in the males in the CAN group was significantly greater than in any other group (Tukey’s test). In the males, the gene expression in the CAN group was greater than in the SOY group (unpaired *t*-test). B. Plasma renin concentration was significantly different between sexes but not between the 2 different diets (two way ANOVA). In the males, plasma renin level in the CAN group was significantly greater than in the SOY group (Tukey’s test and unpaired *t*-test). In the females, plasma renin level in the CAN group was significantly less than in the SOY group (Tukey’s test). C. Plasma angiotensin II concentration was significantly different between sexes and between the 2 different diets (two way ANOVA). In the males, plasma angiotensin II concentration in the CAN group was significantly greater than in the SOY group (Tukey’s test and unpaired *t*-test).

Expression of mRNA for renin in the kidney is shown in Fig. 7A. The gene expression was significantly different between the sexes and between the animals given the 2 different diets (two way ANOVA). In the males, the gene expression in the CAN group was significantly greater than in the SOY group (Tukey’s test and unpaired *t*-test). In contrast, no difference in such expression was found between the dietary groups in the females (Tukey’s test and unpaired *t*-test).

#### 3.2.6 Plasma concentrations of renin and angiotensin II

Plasma concentrations of renin and angiotensin II are shown in Fig. 7B and C, respectively. Plasma renin concentration was significantly different between the sexes but was not different between the animals given the 2 different diets (two way ANOVA). In the males, plasma renin concentration in the CAN group, 624.6 ± 34.8 pg/mL, was significantly higher than in the SOY group, 439.6 ± 16.5 pg/mL (Tukey’s test and unpaired *t*-test). In the females, plasma renin concentration in the CAN group, 193.2 ± 45.6 pg/mL, was significantly lower than in the SOY group, 305.6 ± 77.6 pg/mL (Tukey’s test). Angiotensin II concentration was also significantly different between the sexes and between the animals given the 2 different diets (two way ANOVA). In the males, angiotensin II concentration in the CAN group, 65.33 ± 5.23 pg/mL, was significantly higher than in the SOY group, 48.9 ± 3.42 pg/mL (Tukey’s test and unpaired *t*-test). In contrast, in the females, angiotensin II concentration in the CAN group, 0.61 ± 0.27 pg/mL, and in the SOY group, 1.66 ± 0.42 pg/mL, did not differ significantly.

##### 3.2.6.1 Organ weights and macroscopic findings

Absolute and relative weights of the organs are shown in Table 4. The absolute weights of the brain, heart, lung, spleen, liver and kidney were significantly different between the sexes, and these organs in the males were heavier than in the females (two way ANOVA). The relative weights of the brain, heart, liver, kidney and adrenal gland were also significantly different between the sexes. The relative weights of the heart and kidney in the males were heavier, while those of the brain, liver and adrenal gland were lighter than in the females (two way ANOVA). The relative weights of the heart, liver and kidney were significantly different between the animals given the SOY diet and those given the CAN diet, and the weights were heavier in the animals given the CAN diet (two way ANOVA). In the males, the CAN diet significantly increased the relative weights of the heart and the kidney (Tukey’s test and unpaired *t*-test). In the females, the CAN diet significantly increased the relative weights of the liver (Tukey’s test and unpaired *t*-test). The absolute heart weight was slightly but significantly decreased (unpaired *t*-test), and the absolute liver weight was increased by the CAN diet (unpaired *t*-test). Both absolute and relative uterus weights were decreased in the CAN group (unpaired *t*-test).

**Table 4:** Absolute and relative organ weights in SHRSP of both sexes given SOY or CAN diet for 8 weeks.

Organs		Male						Female					
		SOY			CAN			SOY			CAN		
Brain	Absolute weight (g)^§^	1.97	±	0.02	1.96	±	0.02	1.92	±	0.01	1.92	±	0.01
	Relative weight (mg/g)^§^	7.17	±	0.25	7.76	±	0.40	10.8	±	0.16	11.4	±	0.25
Heart	Absolute weight (g)^§^	1.48	±	0.04	1.52	±	0.04	0.78	±	0.01	0.74	±	0.01*
	Relative weight (mg/g)^§,†,¶^	5.24	±	0.16^b^	5.97	±	0.11^a,*^	4.34	±	0.09^c^	4.38	±	0.06^c^
Lung	Absolute weight (g)^§^	1.34	±	0.20	1.06	±	0.06	0.85	±	0.01	0.85	±	0.01
	Relative weight (mg/g)	4.91	±	0.79	4.07	±	0.08	4.77	±	0.09	5.03	±	0.09
Spleen	Absolute weight (g)^§^	0.62	±	0.02	0.62	±	0.02	0.43	±	0.02	0.41	±	0.02
	Relative weight (mg/g)	2.24	±	0.06	2.41	±	0.09	2.41	±	0.07	2.45	±	0.08
Liver	Absolute weight (g)^§,¶^	8.03	±	0.12^a^	7.95	±	0.21^a^	5.19	±	0.08^b^	5.66	±	0.11^b,*^
	Relative weight (mg/g)^§,†^	28.4	±	0.31^c^	31.2	±	0.47^b^	29.2	±	0.17^c^	33.5	±	0.64^a,*^
Kidneys	Absolute weight (g)^§^	2.36	±	0.04	2.30	±	0.04	1.31	±	0.02	1.29	±	0.03
	Relative weight (mg/g)^§,†^	8.34	±	0.09^b^	9.08	±	0.25^a,*^	7.33	±	0.06^c^	7.66	±	0.15^c^
Adrenal glands	Absolute weight (mg)	48.9	±	2.57	55.0	±	2.78	51.3	±	1.13	49.4	±	0.95
	Relative weight (mg/g)^§^	0.17	±	0.01	0.21	±	0.02	0.29	±	0.01	0.29	±	0.01
Testes	Absolute weight (g)	2.98	±	0.04	2.86	±	0.08		–			–	
	Relative weight (mg/g)	10.6	±	0.21	11.3	±	0.34		–			–	
Epididymides	Absolute weight (g)	0.98	±	0.02	0.93	±	0.03		–			–	
	Relative weight (mg/g)	3.49	±	0.09	3.64	±	0.06		–			–	
Ovaries	Absolute weight (mg)		–			–		66.2	±	1.68	68.5	±	2.38
	Relative weight (mg/g)		–			–		0.30	±	0.01	0.29	±	0.01
Uterus	Absolute weight (g)		–			–		0.39	±	0.04	0.29	±	0.01*
	Relative weight (mg/g)		–			–		2.20	±	0.20	1.71	±	0.05*

SOY, 10 w/w% soybean oil diet (control); CAN, 10 w/w% canola oil diet.

Values are means ± SEMs of 10 animals.

^§^ and ^†^, Significantly different between sexes and between diets, respectively (p<0.05, two way ANOVA).

^¶^, Interaction exists between the factors, sexes and diets (p<0.05, two way ANOVA).

Identical alphabetical letters (above SEMs) indicate the absence of significant differences between the groups (p>0.05, Tukey’s test).

*, Significantly different from the SOY group in each sex (p<0.05, unpaired *t*-test).

**Table 5:** Microscopic findings in SHRSP of both sexes given SOY or CAN diet for 8 weeks.

Organs	Findings	Male		Female	
		SOY	CAN	SOY	CAN
Brain	Infarction or laminar necrosis of cortex	2	3	0	0
	Glial reaction, microglia or astroglia	1	1	0	0
	Degeneration of corpus callosum	1	0	0	0
	Petechial hemorrhaging	4	0	0	0
Heart	Myocardium degeneration	3	7	0	0^#^
	Perisclerosis of arterioles	5	9	0^‡^	0^#^
	Thrombosis in arterioles	3	7	0	0^#^
Lung	Alveolar hemorrhage/congestion	3	3	0	0
	Macrophage infiltration	2	1	0	0
Spleen	White pulp atrophy	1	2	0	0
	Arterial wall thickening	6	7	0^‡^	0^#^
Liver	Centrilobular single cell necrosis	5	7	0^‡^	0^#^
	Microgranuloma	5	6	1	0^#^
	Periportal fatty change	6	3	7	2
	Perisclerosis of arterioles	0	3	0	0
Kidney	Basophilic tubule	10	10	0^‡^	2
	Hyalin cast	5	9	0^‡^	0^#^
	Glomerular degeneration	9	10	1^‡^	0^#^
Testis	Lymphocyte perivascular infiltration	0	3	–	–
	Seminiferous tubule atrophy	0	1	–	–
	Leydig cell atrophy	0	1	–	–
Epididymis	Cell debris in lumen	0	5*	–	–
Uterus	Squamous cell metaplasia	–	–	1	0

SOY, 10 w/w% soybean oil diet (control); CAN, 10 w/w% canola oil diet.

Values are means ± SEMs of 10 animals.

‡, Significantly different from the male animals given SOY diet.

#, Significantly different from the male animals given CAN diet.

*, Significantly different from the SOY group in the male animals (p<0.05, Fisher’s exact probability test).

##### 3.2.6.2 Microscopic findings

In the males, most of the organs examined showed histological abnormalities. However, the frequencies of the abnormalities were not significantly different between the 2 dietary groups, except the increased cell debris in the lumen of the epididymis ducts in the CAN group (Table 5). In contrast, the organs of the females did not show any abnormal findings, except in the liver and the kidney. One animal in the SOY group showed micro-granulomata, and 7 animals in the SOY group and 2 animals in the CAN group showed periportal fatty changes in the liver. One animal in the SOY group showed glomerular degeneration, and 2 animals in the CAN group showed basophilic tubules in the kidney. The heart in the males tended to be injured compared with the heart in the females. The sex-differences were more remarkable between the CAN groups than between the SOY groups.

## 4 Discussion

The present study investigated the influence of a CAN diet on the pathophysiology in SHRSP of both sexes for the first time. In the male animals, the 8-week feeding with a CAN diet gave rise to many adverse events, as follows: increases in blood pressure and heart rate; insulin resistance; thrombopenia; injuries or abnormalities in renal and cardiac pathology; reduction in plasma testosterone, with a concomitant increase in plasma aldosterone; and increases in the expression of mRNA for renin in the kidney and in plasma concentrations of renin and angiotensin II. These findings should be related to the aggravated metabolic syndrome-like conditions and the consequently shorter life. In contrast, the female animals on the CAN diet showed neither the adverse events noted above, except the decrease in testosterone, nor a significant shortening of life. These results demonstrate the differences between sexes in the adverse effects or toxicity of CAN in SHRSP.

In the CAN group, BW gain declined in both, the male and the female animals, starting at around the 60th and 80th day, respectively, at which times the first animals with stroke-related symptoms appeared. Thereafter, BW was consistently lower in the CAN group than in the SOY group, irrespective of sex. BW of the males in the CAN group decreased day by day and became significantly less than that in the SOY group between the 238th and 308th days. The decrease in BW may be due to age-related, pronounced metabolic syndrome-like conditions, including hypertension. In contrast, the females given the CAN diet never showed such an evident decrease in BW. These findings demonstrate that while the CAN diet has detrimental effects on SHRSP regardless of sex, the effects are more pronounced in males. As for FC during the first several weeks, daily intake of the CAN diet tended to be greater than that of the SOY diet. Such a trend was found in both sexes but was clearer in the male animals. It was reported that a diet containing CAN, compared with one containing SOY, increased locomotor activity (ambulation and rearing counts and wheel cage performance), shortened transfer latency in the elevated plus maze and lowered the pain threshold in male mice [26]. Also, in the present study, the animals given the CAN diet appeared to be restless and irritable, during daily handling, compared with those in the SOY group (data not shown). SHR and SHRSP are known to show hyperactivity [27,28], and SHRSP is used as a model animal for attention-deficit/hyperactivity disorder [29,30], so spilling the powdered diet due to the increased locomotion probably resulted in an apparent increase in FC in the CAN groups. The apparent increase in FC during the early several weeks and the ensuing suppression of BW gain in the CAN group were common in both sexes. Thus, the changes may be associated with the detrimental effects of CAN, which promotes the hyperactivity, but that may not be directly connected to aggravation of the SHRSP-specific diseases which led to life-shortening.

In the previous study, it was found that systolic blood pressure of male SHRSP was elevated by a 4-week ingestion of a CAN diet and was significantly higher than that in animals given a SOY diet [7]. In the present study, a CAN-induced elevation of blood pressure was confirmed in the male animals. The increase in blood pressure was not accompanied by reflex bradycardia, but by tachycardia, suggesting some sympathomimetic action of the CAN diet. It was also found in the previous study that noradrenaline-constriction in perfused mesenteric vascular bed isolated from male SHRSP given CAN for 4 weeks by gavage tended to be suppressed, compared to the response in the animals given SOY [6]. A significant suppression of the noradrenaline-constriction of isolated perfused mesenteries was also observed in SHR fed a CAN diet for 26 weeks, compared with the control animals fed a SOY diet [32]. Although peripheral catecholamine concentrations were not determined and the activation of adrenoceptor mediated signal transduction was not confirmed, at least, in male SHRSP, CAN ingestion might stimulate sympathetic nerves, and a resultant down-regulation of adrenergic response might occur in the peripheral vasculature. As described later, the CAN-induced increase in aldosterone concentration should be considered as a possible mechanism for the sympathetic drive.

In OGTT, the time-concentration curves for serum insulin in the 2 dietary groups were the same for both sexes, whereas the plasma Glu concentration in the male animals in the CAN group was consistently higher than in the SOY group. The Glu level in the female animals given the CAN diet was also tended to be higher than in those given the SOY diet, but the difference was not significant. These findings suggest that the suppression by CAN of peripheral Glu uptake per se is common in SHRSP of both sexes, but the suppression becomes evident only in males. It was reported that the decreased number of insulin receptors and blunted Glu transport in orchiectomized rats were restored by testosterone replacement [33]. Further, testosterone is reported to increase Glut-4 expression in the skeletal muscle and Glu uptake *via* the Glut-4 in neonatal cardiomyocytes of the rat [34,35]. Thus, it is conceivable that the decreased level of testosterone caused by the CAN diet led to insulin resistance in male SHRSP. In the female animals, the CAN diet also decreased plasma testosterone concentration, with a concomitant decrease in estradiol. However, these changes in sex hormones were not accompanied by any evident changes in plasma Glu concentration. Although it is still controversial how estrogen affects Glu uptake in the peripheral organs of the rat [[36], [37], [38], [39], [40]], the CAN-induced decreases in sex hormones hardly affected the peripheral Glu uptake in female SHRSP.

Previously, CAN-induced thrombopenia in male rats of WKY [31,32,41], SHR [32] and SHRSP strains [8] was reported. Thrombopenia was found in newborn piglets given a formula containing CAN, as well [[42], [43], [44]]. Kramer et al. [43] reported that the decrease in platelet count was accompanied by a longer bleeding time, and they gave a possible causative, nervonic acid, which is a metabolite by a fatty acid elongase from erucic acid, which occurs in rapeseed oil and CAN. They used piglets of both sexes but did not note a difference between sexes in the results. In the present study, a significant decrease by the CAN diet of the platelet count was confirmed in SHRSP, and it was limited to the male animals. Nervonic acid was not measured, and it is, thus, unclear whether nervonic acid synthesized from erucic acid also has relevance to the thrombopenia in male SHRSP. On the other hand, Tomita et al. [45] reported age- and blood pressure-dependent facilitation of serotonin degranulation from platelets in SHRSP of both sexes compared to that in the normotensive counterpart strain, WKY, indicating that the longer the hypertension continues, the more strongly the platelets are activated. They also reported that the hypertension state decreased platelet count and increased degranulation when they compared male SHRSP with and without stroke [46]. In the present study, aggravation of hypertension in the CAN group, compared to the SOY group was found in the male animals, while the female animals in the 2 dietary groups showed comparable blood pressure. Therefore, the decrease of platelet count in the male animals by the CAN diet might be caused *via* the activation and increased consumption of platelets in the injured peripheral vasculature due to the aggravated hypertension.

The postmortem examination in the survival experiment revealed hemorrhaging, thrombosis and edema in the brain, hypertrophy, myocardial fibrosis and atherosclerosis in the heart, and chronic nephrosis in almost all the animals used, suggesting that they were dying of hypertension-related complications specific to SHRSP. Moreover, the abnormal findings other than those related to the genetic diseases in SHRSP could not be found, and there was no difference between the groups in the organ weight or the incidence of pathological abnormalities, regardless of sex. These findings corroborated the fact that there were no pathological abnormalities particular to the animals given the CAN diet, but the CAN diet worsened the genetic diseases, that is, the metabolic syndrome-like conditions, including hypertension, in SHRSP, especially in the male animals.

In the 8-week ingestion study, the pathological examination of the kidney showed basophilic tubules, hyalin casts and glomerular degeneration in the male animals, but the frequency of each finding was similar in the 2 dietary groups. Thus, the renal tissue injury itself was thought to be the predominant and inevitable pathophysiology of male SHRSP, which occurs age-dependently during the progression of the genetic disease specific to the strain. Moreover, it does not necessarily have direct relevance to the life-shortening caused by the CAN diet in the male animals because no difference between the sexes was found in the survival of the animals given the SOY diet, even though the female animals at the 8th week of ingestion did not show any abnormalities in the kidney, in contrast to the male animals, in which renal injury was clearly evident and was similar to that in the male animals given the CAN diet. The greater relative weight of the kidney in the male animals in the CAN group than in the SOY group could be explained by the accelerated aggravation of hypertension caused by the CAN diet, considering the similar relative kidney weights in the 2 dietary groups at natural death or after euthanasia in the survival study. In the heart of the male animals in the CAN group, thrombosis in and perisclerosis of the arterioles, and myocardial degeneration tended to be frequenter than in the SOY group. These findings, together with the significantly greater relative heart weight, suggest that higher blood pressure adversely affected the male animals in the CAN group. Putting these pathological findings together, the increase in blood pressure should be the cause of the life-shortening. In fact, in the 8-week ingestion study, the absolute heart weight in the female animals in the CAN group was decreased slightly but significantly, compared to that in the SOY group. This might be related to the tendency of lowered blood pressure and heart rate in the female animals in the CAN group than in the SOY group. The decreases in the absolute and relative uterus weights in the CAN group, compared to the SOY group, might reflect the lower level of plasma estradiol in the CAN group. Diversity of estrous cycles is also a possible explanation because the estrous cycles of the animals had not been synchronized. Increased cell debris in the seminiferous tubular lumen was the only abnormal outcome in the male animals in the CAN group. That finding, together with the significant decrease in testosterone level, suggests that the testis is at least one of the target organs of CAN toxicity.

It has been reported in male SHRSP that plasma lipids, TCh, FCh, TG FFA and phospholipid tended to be increased after the 8-week ingestion of the CAN diet [8] and that plasma TCh and LDLC levels were increased after a 25-day ingestion of the CAN diet [10]. Increases in blood lipids caused by a CAN diet were also reported in male rats of strains other than SHRSP: in WKY rats after a 13-week ingestion [31], and in WKY rats and SHR after a 26-week ingestion [32,47]. Since dyslipidemia has been regarded as one of the predisposing factors for atherosclerosis in metabolic syndrome in humans [48], the elevated plasma lipids could be suspected as the cause of the life-shortening in SHRSP, a model animal for metabolic syndrome (see the Introduction, above). However, the significant increases in plasma lipids and relative liver weight were not specific to the male animals but were also found in the female animals, which did not show any adverse events caused by the CAN diet. Pathological findings in the liver showed a sex difference, but there were no differences between the groups. Therefore, the increased plasma lipids are attributable to the CAN diet but have no connection with the pathological changes or with aggravation of the hypertension-related complications which lead to the life-shortening in male SHRSP.

The expression of mRNA for renin and plasma concentrations of renin and angiotensin II were significantly increased in the male animals fed the CAN diet, compared with those fed the SOY diet. Thus, the activated renin-angiotensin-aldosterone system (RAAS) is undoubtedly associated with the aggravation of hypertension in the male animals. Previously, Miyazaki et al. [49] reported an increased expressions of mRNA for renin, TGF-β and fibronectin in male SHRSP given a CAN diet for 9 weeks, compared to those given a SOY diet, and noted the renal injury caused by the CAN diet. If the renal injury, with consequent activation of RAAS, was responsible for the life-shortening, the CAN group should have shown severer renal lesions than the SOY group in the present study. However, at the 8th week of ingestion, the renal lesions were comparable in the 2 groups. In contrast, the cardiac injuries in the male animals in the CAN group tended to be more frequent than in the SOY group at the same time point. Therefore, the elevation of blood pressure *via* sympathetic drive or renin release owing to sympathetic nerve stimulation, or both, is presumed to trigger the life-shortening. The functional changes appeared to have no connection with the histological or structural changes in the kidney of SHRSP, at least under the present experimental conditions. Thus, the question remains of how the CAN diet caused a sympathetic drive or renin release.

Previously it was found that the plasma aldosterone level was significantly higher in male Wistar rats fed a CAN diet for 10 weeks than in animals fed a SOY diet [50]. Moreover, it was also found that testosterone levels in the testis and plasma in SHRSP were significantly decreased after ingestion of a CAN diet for 12 weeks, compared to the control animals given a SOY diet [12]. In the present study, a decrease in plasma testosterone and an increase in plasma aldosterone were observed concomitantly in male SHRSP after an 8-week ingestion of the CAN diet, compared to those given the SOY diet. According to Kau et al. [51], plasma aldosterone level in male rats was increased after orchidectomy and was restored by testosterone replacement. They also reported that testosterone in the range of 10^−9^–10^−7^ mol/L concentration-dependently inhibited aldosterone production in primary cultured zona glomerulosa (ZG) cells from the rat. These findings are suggestive of the existence of a physiological negative regulation by testosterone of aldosterone production in male rats and of the inhibition by the CAN diet of that negative regulation *via* the suppression of testosterone production in the testis. In other words, plasma testosterone may negatively regulate the production of aldosterone at the adrenal gland in a physiologically mutual relation. So, the decrease in the level of testosterone by CAN would inhibit the negative regulation of the production of aldosterone. The resultant increase in aldosterone level would then trigger the hypertension and vascular injury. Aldosterone is known to stimulate MR at the rostral ventrolateral medulla and activates the sympathetic nervous system [[52], [53], [54]]. The enhanced sympathetic tonus may directly elevate blood pressure and heart rate and then stimulate the renin release from the juxta glomerular apparatus. Although abnormalities in the testicular pathology could not be found, the increased cell debris in the epididymis might reflect a disorder of testicular sperm formation due to the reduced testosterone production caused by the CAN diet. The significant decrease in plasma testosterone with a resultant decrease in estradiol was also observed in the female animals fed the CAN diet. There are no reports about the regulation by testosterone of aldosterone production in female rats. Although estrogen is reported to reduce aldosterone production [55,56], neither the decreased testosterone nor the decrease in estradiol appeared to affect aldosterone level in the female animals given the CAN diet. Thus, the present results indicate that at least testosterone has no role in the regulation of aldosterone production in the adrenal gland in female SHRSP. The basal level of plasma aldosterone was higher in the female animals than in the male animals. Such a difference is in line with the previously reported sex difference in plasma aldosterone level in SHR [57] and in Wistar rats [[58], [59], [60]]. The decreased testosterone led to a significant facilitation of aldosterone production in the male animals but not in the female animals. Such a regulation should be unique to male animals and might play a crucial role in the sex difference of CAN diet-induced adverse events.

## 5 Conclusion

The present study clearly demonstrated sex differences in CAN toxicity in SHRSP for the first time. The lifespan of the males in the CAN group was clearly shorter than in the SOY group, whereas that in the females did not show any differences between the groups. The males fed the CAN diet showed significantly increased blood pressure, thrombopenia and insulin-tolerance, all of which are included in major components of the symptoms in metabolic syndrome. However, those parameters did not show any differences between the 2 dietary groups in the females. Although plasma testosterone level was significantly lower in the animals of both sexes fed the CAN diet than those fed the SOY diet, the decreased testosterone was accompanied by a significantly increased plasma aldosterone only in the males. Thus, it is suggested that the sex differences may be attributable to the increased aldosterone level, which occurred only in the males, and which triggers the aggravation of the metabolic syndrome-like conditions. The CAN diet tended to decrease BW, shorten survival time and suppress Glu uptake in peripheral tissues, and increased plasma lipids in the females, as well. However, these effects, except the increased plasma lipids, were not significant when compared to those in the females given the SOY diet. Interestingly, there is a possibility that testosterone negatively regulates aldosterone production in the physiology of the males, and the inhibition of that regulation caused by CAN ingestion led to the adverse events.

## 6 Limitations of the present study

The changes by the CAN diet in plasma testosterone and aldosterone concentrations were clearly shown in the present study. These changes were sufficient to explain the possible involvement of these steroids, at least as triggers, in the adverse events caused by CAN in male SHRSP. However, it would have been helpful and persuasive to provide information about the expressions of the genes and proteins responsible for production of testosterone and aldosterone. Accordingly, work in the authors’ laboratory is currently ongoing to investigate changes in the steroid hormone levels and the expressions of mRNA and of proteins for several enzymes in the metabolism pathway of steroids. In addition, whether or not an MR antagonist ameliorates the adverse events in the males was not examined, which could have provided direct evidence for the involvement of aldosterone, although such an antagonism cannot separate the cause, sympathetic drive, and the effect, the resultant activation of RAAS by the CAN diet in the males.

In the present study, no causative factors of CAN toxicity could be examined for adverse effects, since no candidate factors have been identified yet. Some ingredients in CAN have been referred to as possible causatives: In 1975, Minetoma et al. [61] reported that rapeseed meal caused thyroid hypertrophy in laying hens and that the amounts of isothiocyanate and oxazolidinethione, catabolic products of glucosinolate in the meal, were proportional to the hypertrophy. Thereafter, in 1996, Huang et al. [1] investigated the effects of the sulfur-containing compounds, butyl, phenethyl and allyl isothiocyanate at concentrations similar to those in CAN and found that none of those substances affected the survival of SHRSP. In 1998, Miyazaki et al. [3] indicated that the adverse factors were other than fatty acids unique to CAN, since the fatty acid fraction isolated by lipase-treatment of CAN did not shorten the survival of SHRSP. In 2000, Ratnayake et al. noted that increased phytosterols in plasma or tissue by ingestion of CAN might make membranes fragile either directly or by replacing cholesterol and result in hemorrhagic injuries [5]. This explanation is plausible, and the amount of phytosterols appeared critical to the life-shortening effects of CAN. However, in the same paper, it was reported as an exception to the “phytosterol theory” that olive oil, which contains only one third of the phytosterols in CAN, revealed life-shortening effects comparable to CAN. Moreover, other researchers [3,8,62] have reported that there was no correlation between phytosterol content and survival time of SHRSP for several other vegetable oils. Recently dihydro-vitamin K1, which interferes with vitamin K2 effects, and reveals various adverse effects including endocrine disruption, and some substances with similar activity to dihydro-vitamin K1 were also proposed as potential stroke-stimulating factors in CAN [63]. Thus far, decisive causatives for CAN toxicity have not yet been identified. However, it should be worthwhile to make an effort to gather information about CAN toxicity based on precise research in order to find clues to identifying those causatives.

## Funding sources

This study was supported by 10.13039/501100001691Japan Society for the Promotion of Science (Grant-in-Aid for Scientific Research (C) #26350133), Aichi Health Promotion Foundation and by Nagono Foundation for Promotion of Medical Sciences.

## Conflict of Interest

The authors declare no conflict of interest.

## CRediT authorship contribution statement

**Mai Nishikawa:** Conceptualization, Methodology, Investigation, Validation, Writing - original draft. **Naoki Ohara:** Conceptualization, Methodology, Validation, Writing - review & editing, Project administration, Supervision. **Yukiko Naito:** Methodology, Investigation, Validation, Supervision. **Yoshiaki Saito:** Methodology, Investigation, Validation, Supervision. **Chihiro Amma:** Methodology, Investigation, Validation, Writing - review & editing. **Kenjiro Tatematsu:** Conceptualization, Writing - review & editing, Supervision. **Jinhua Baoyindugurong:** Methodology, Investigation, Validation, Writing - review & editing. **Daisuke Miyazawa:** Methodology, Investigation, Validation, Supervision. **Yoko Hashimoto:** Methodology, Investigation, Validation, Writing - review & editing. **Harumi Okuyama:** Conceptualization, Supervision.

## Declaration of Competing Interest

The authors report no declarations of interest.
